# Evaluation of a single-dose of intravenous magnesium sulphate for prevention of postoperative pain after inguinal surgery

**DOI:** 10.4103/0019-5049.76605

**Published:** 2011

**Authors:** Shashi Kiran, Rachna Gupta, Deepak Verma

**Affiliations:** Department of Anaesthesiology and Critical Care, Pt. B D Sharma University of Health Sciences, Rohtak, India

**Keywords:** Intravenous, magnesium sulphate, postoperative pain

## Abstract

This study was undertaken to study efficacy of single dose of intravenous magnesium sulphate to reduce post-operative pain in patients undergoing inguinal surgery. One hundred patients undergoing inguinal surgery were divided randomly in two groups of 50 each. The patients of magnesium sulphate group (Group-I) received magnesium sulphate 50 mg/kg in 250 ml of isotonic sodium chloride solution IV whereas patients in control group (Group-II) received same volume of isotonic sodium chloride over 30 minutes preoperatively. Anaesthesia was induced with propofol (2 mg/kg) and pethidine (1 mg/kg). Atracurium besylate (0.5 mg/kg) was given to facilitate insertion of LMA. Pain at emergence from anaesthesia and 2, 4, 6, 12 and 24 hours after surgery was evaluated. The timing and dosage of rescue analgesic during first 24 hrs after operation was noted. Pain in postop period was significantly lower in magnesium sulphate group in comparison to control group at emergence from anaesthesia and 2, 4, 6, 12 and 24 hrs postop [1.86 vs. 1.96 (*P*=0.138), 1.22 vs. 1.82 (*P*=0.001), 1.32 vs. 1.88 (*P*=0.000), 2.74 vs. 3.84 (*P*=0.000), 1.36 vs. 2.00 (*P*=0.000) and 0.78 vs 1.30 (*P*=0.000), respectively]. Patients in group-I were more sedated as compared to group-II [sedation score 1.86 vs. 1.40 (*P*=0.000)]. Rescue analgesia requirement postoperatively in first 4, 8 and 16 hrs was significantly lower in patients of group-1 than in group- II [1.9 vs. 3.8 (*P*<0.05), 25.50 vs. 52.50 (*P*<0.05) and 0.000 vs. 7.5 (*P*<0.05)]. Preoperative magnesium sulphate infusion decreases postop pain and requirement of rescue analgesia.

## INTRODUCTION

The concept of pre-emptive analgesia was introduced in by Woolf who demonstrated through experimental studies that postinjury pain hypersensitivity results via a central mechanism. Pre-emptive analgesia has been defined as an antinociceptive treatment that prevents establishment of altered central processing of afferent input from injuries. Therapies that have been tested in pre-emptive trials include NSAIDS, intravenous opioids, intravenous ketamine, peripheral local anaesthetics, caudal and epidural analgesia, dextromethorphan and gabapentin.[1] One intravenous adjuvant medication that has shown potential in pre-emptive analgesia is magnesium.

Magnesium (Mg) is the fourth most common cation in the body and it activates approximately 300 enzymes systems, including most of the enzymes involved in energy metabolism and nucleic acid synthesis. Magnesium is of importance in anaesthesia practice for several reasons. First, the ion is essential for many biochemical reactions and its deficiency may produce clinically important consequences during anaesthesia or in intensive care unit. Second, the extensive use of magnesium sulphate in obstetric practice requires that anaesthesiologists be familiar with the pharmacological action of this drug and its interaction with anaesthetic agents. Third, few of its properties may be of value in certain areas of anaesthetic practice.[2,3]

Various studies have been done regarding role of magnesium sulphate in postoperative analgesia. Since in literature there is no convincing evidence to support analgesic efficacy of magnesium sulphate, therefore it was planned to study the role of magnesium sulphate for postoperative analgesia.

## METHODS

After hospital ethics committee approval a total of 100 patients (15-50 years) of either sex belonging to ASA physical status I or II, were allocated randomly to the magnesium sulphate or the control group. Patients with impaired renal or hepatic function, varying degree of heart blocks, hypertension, neurological disorders, myopathy, diabetes, drugs or alcohol abuse, pregnant women, obese patients (body mass index more than 30 kg/m) and patients treated with calcium channel blockers or magnesium were not included in the study. The patients were randomized to receive either magnesium sulphate 50 mg/kg in 250 ml of isotonic sodium chloride solution IV (Group I) or same volume of isotonic sodium chloride solution(Group XI). Randomization and sample population were derived by using computer-generated Microsoft excel programme. An independent anaesthesiologist who did not participate in the study prepared study medication. The purpose, protocol of study and use of visual analogue scale (VAS) was explained to patients.

The patients were premedicated with tablet alprazolam 0.25 mg the night before and 2 hrs before surgery. Study medication was infused over 30 minutes before induction of anaesthesia. Pulse rate and blood pressure were monitored at 10, 20 and 30 minutes interval during this period. Upon arrival in operating room usual monitoring was established. General anaesthetic was administered using propofol (2 mg/kg) and pethidine (1 mg/kg) for induction. Atracurium besylate (0.5 mg/kg) was given to facilitate insertion of appropriate size of LMA. Anaesthesia was maintained with isoflurane and nitrous oxide in oxygen (FiO_2_=0.4). During intraoperative period any episode of hypotension and/or bradycardia was noted. Postoperatively residual neuromuscular blockade was reversed by using neostigmine (0.05 mg/kg) and glycopyrrolate (0.02 mg/kg).

Pain at rest was evaluated using a 0-10 cm VAS (0 - No pain at all to 10 - Worst pain imaginable) at emergence from anaesthesia and 2, 4, 6, 12 and 24 hrs after surgery. During first 4 hrs the patients were kept in recovery room and rescue analgesia was provided at VAS≥3 in the form of pethidine 0.5 mg/kg IV. If VAS score did not decrease to <3, pethidine 0.25 mg/kg IV repeated with a maximum dose of 1 mg/kg hr. Sedation was monitored by using a four-point rating scale (1 – patient fully awake, 2 – patient somnolent but responds to verbal commands, 3 – patient somnolent but responds to tactile stimulation, 4 – patient asleep but responds to pain).

Thereafter, the patients were sent to ward and diclofenac sodium 75 mg intramuscularly was given on demand. The timing and dosage of rescue analgesic and total consumption of diclofenac sodium during first 24 hrs after operation was noted.

### Statistical analysis

All the data were compiled and continuous variables were analysed using Student t-test. Scores were analysed using the Mann Whitney U-test for independent samples. Differences among the group means were compared using analysis of variance or paired t-test. Dichotomous data were analysed using χ^2^ test. A ‘*P*’ value of <0.05 was considered significant, <0.001 highly significant and >0.05 was considered insignificant.

## RESULTS

**Figure 1:**
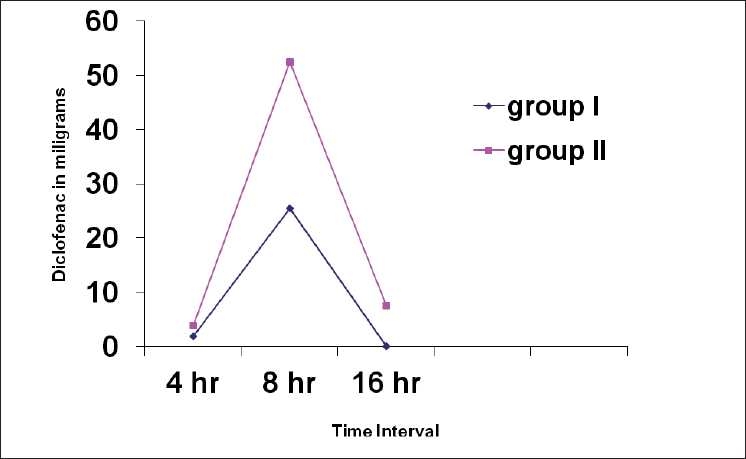
Rescue analgesic requirment (diclofenac) in recovery room at 4hr, 8hr and 16hr

The demographic profile and duration of surgery of patients in two groups were comparable. Comparison of haemodynamic paremeters during study medication and intraoperative period between group I and group II at different time intervals was statistically insignificant. At different time intervals patients in group I had less pain than group II when compared on VAS (*P*<0.05) except at emergence of anaesthesia (*P*>0.05) [Table 1]. Patients in group-I were more sedated as compared to group II patients (*P*<0.01) [Table 2]. Rescue analgesia requirement of patients in first 4 hrs in recovery room and at 8 hrs and 16 hrs in surgical ward was less in group I than in group II (*P*<0.05)) [Figure 1]. None of patients demonstrated bradycardia, hypotensive episodes, hypoxia or hypoventilation during intraoperative as well as in postoperative period in recovery room.

**Table 1:** Assesment of pain in postoperative period (Visual analogue scale) (1-10)

	Group I (Mean±SD)	Group II (Mean±SD)	*P* value
Emergence of anaesthesia	1.86±.70	1.96±0.53	0.138
After 2 hrs	1.22±.76	1.82±96	0.001
4 hrs	1.32±.84	1.88±.44	0.000
8 hrs	2.74±1.43	3.84±1.46	0.000
16 hrs	1.36±.69	2.00±.76	0.000
24 hrs	0.78±.68	1.30±.46	0.000

‘*P*’<0.05 significant, ‘*P*’>0.05 insignificant

**Table 2:** Postoperative sedation score in recovery room

Group	Sedation score (mean ± SD)	*P* value
Group-I (n=50)	1.86±0.64	
Group-II (n=50)	1.40±0.49	0.000

## DISCUSSION

Our study has shown that infusion of magnesium sulphate (50 mg/kg) given before induction of anaesthesia is associated with less postoperative pain in patients undergoing inguinal surgery.

Previous studies investigating the analgesic efficacy of magnesium sulphate have shown conflicting results. Tramer and others observed that pretreatment with IV magnesium sulphate had no impact on postoperative pain and analgesic consumption, but the patients in their study received only diclofenac suppository immediate preoperatively.[4] Since intraoperative magnesium is known to potentiate the analgesic efficacy of opioids, the administration of intraoperative pethidine resulted in superior pain relief in our patients. Moreover, in their study all patients undergoing hernia repair had an ilioinguinal and iliohypogastric nerve block done with 20 ml of 0.5% bupivacaine at the end of surgery resulting in consistently decreased pain scores in first 4 hrs. Similarly, Ko and others have also discounted the efficacy of magnesium sulphate administration on postoperative analgesic requirement, but they had also used epidural analgesia in their study.[5] It is possible that superior analgesic efficacy of nerve block or epidural analgesia in their patients might have masked analgesic efficacy of magnesium sulphate. No nerve block or epidural analgesia was used in any of our patients. Dabbagh and others observed that magnesium sulphate can serve as a supplementary analgesic leading to less morphine consumption in postoperative period in patients undergoing orthopaedic surgery under spinal anaesthesia.[6] The role of nitric oxide in potentiation of magnesium on morphine analgesia was discussed by Karaaslan and Arslan.[7]

Levaux and others, Seyhan and others and Ryu and others. have also reported that magnesium sulphate boluses were effective for postoperative pain relief after orthopaedic and gynaecological surgery, respectively.[8–10] However, they had used continuous infusion or repeat bolus in addition to initial bolus of magnesium sulphate in their studies in contrast to single bolus preoperatively in the present study. Efficacy of magnesium sulphate in reducing postoperative pain following laparoscopic cholecystectomy and coronary artery bypass grafting was observed by other investigators as well.[11,12] We administered magnesium sulphate in dosage of 50 mg/kg IV infused over 30 min before induction of anaesthesia without any subsequent infusion. This dosage has been reported to be safe without any adverse effects as reported by several workers. It has been suggested that NMDA blocking drugs should be given before beginning of nociceptive stimulus to inhibit process of central sensitization.[13]

Mechanism of analgesic effect of magnesium sulphate is not clear but inhibition of calcium channels and NMDA receptors seem to play an important role. It has been commented by various workers that calcium channel blockers have an antinocieptive action in algesiometric tests in rats under acute conditions.[14] Sanitillian *et al*. studied the effect of nimodipine on enhancement of opiate analgesia in cancer patients and found that calcium channel blockers can enhance the opioid analgesia in patients chronically treated with morphine.[15] The analgesic action of calcium channel blockers could be mediated by an increase of the nociceptive threshold resulting from interference with calcium influx because the latter is important for the release of neurotransmitters and other substances implicated in nociception and inflammation.

Woolf *et al*. studied the dependence of the central sensitisation on NMDA receptor activation in rats and found that NMDA receptor activation is involved in the induction and maintenance of central sensitization processes that characterize post injury pain states.[16] Therefore, NMDA receptor antagonist may play a role in prevention and treatment of perioperative pain. Thompson *et al*. found that IV magnesium sulphate produced a dose-dependent reduction in halothane minimum alveolar anaesthetic concentration (MAC), as measured by the tail-clamp technique, which could be considered as an anaesthetic effect in an acute pain model.[17]

It is well known that magnesium sulphate inhibits acetylcholine release at motor nerve terminals, thus potentiating the effect of neuromuscular blocking agents. In some studies, prior administration of magnesium sulphate prolonged clinical duration of intermediate acting non-depolarizing neuromuscular blocking agents.[18,19] In our study, we did not monitor neuromuscular block by train of four method; nevertheless no clinical prolongation of neuromuscular block was observed with magnesium sulphate. This reason could be attributed to use of atracurium in our study. Moreover, we used magnesium sulphate only as single bolus dose whereas most of the studies have used magnesium sulphate as subsequent infusion also in addition to initial single bolus.

One limitation in our study was that we did not measure serum magnesium and cerebrospinal fluid magnesium concentration. However, it has been studied that most of total body magnesium (99%) is intracellular and estimation of plasma magnesium does not represent magnesium content of body tissues. Therefore, there is lack of correlation between plasma magnesium concentration and total body magnesium content.

It is well known that magnesium may induce hypotension directly by vasodilatation as well as indirectly by sympathetic blockade and inhibiton of catecholamine release. However, we did not observe any hypotensive episode in our patients treated with magnesium sulphate. Transient fall in blood pressure was observed in our patients at induction in both groups which can be attributed to use of propofol as an induction agent. None of our patients had any significant bradycardia that required treatment. However, significant bradycardia has been reported by some of the authors, including one study attributing it to over use of propofol aiming to maintain bispectral index within the target values.[4,13] Other authors assessing role of magnesium sulphate in postoperative pain relief have not commented upon heart rate changes with magnesium sulphate.[8,20]

In our study patients receiving magnesium sulphate were found to be more sedated in immediate postoperative period as compared to control group, although they were easily arousable. This is expected as magnesium is regarded as a CNS depressant. Many studies evaluating the effect of magnesium sulphate in postoperative analgesia have not measured the postoperative sedation scores.[4,5,8,10] These results are different from the studies where patients receiving magnesium sulphate were not observed to be sedated in spite of using magnesium sulphate as bolus and/or infusion.[13,20]

## CONCLUSIONS

Administration of intravenous magnesium sulphate 50 mg/kg preoperatively significantly reduces postoperative pain in patients undergoing inguinal surgery. This finding complements the known use of magnesium in anaesthesia as an antiarrhythmic or anticonvulsant drug. In this limited number of patients we did not find any evidence of adverse effect owing to magnesium sulphate. However, further studies should be done regarding different dosages of magnesium and comparison with established analgesic drugs and other routes of administration of magnesium sulphate such as (intrathecal, epidural and as adjuvant with local anaesthetic for regional nerve blocks). More studies should also be done to establish a dose-response relationship for potential antinociceptive effects of magnesium.
